# New Viruses in Idiopathic Human Diarrhea Cases, the Netherlands

**DOI:** 10.3201/eid2007.140190

**Published:** 2014-07

**Authors:** Saskia L. Smits, Claudia M.E. Schapendonk, Janko van Beek, Harry Vennema, Anita C. Schürch, Debby Schipper, Rogier Bodewes, Bart L. Haagmans, Albert D. M. E. Osterhaus, Marion P. Koopmans

**Affiliations:** Viroclinics Biosciences, Rotterdam, the Netherlands (S.L. Smits, A.D.M.E. Osterhaus);; Erasmus Medical Center, Rotterdam (S.L. Smits, C.M.E. Schapendonk, J. van Beek, A.C. Schürch, D. Schipper, R. Bodewes, B.L. Haagmans, A.D.M.E. Osterhaus, M.P. Koopmans);; National Institute for Public Health and the Environment, Bilthoven, the Netherlands (J. van Beek, H. Vennema, M.P. Koopmans)

**Keywords:** diarrhea, idiopathic human diarrhea cases, viruses, circular DNA virus, circovirus, picobirnavirus, bufavirus, enteric infections, random amplification, next-generation sequencing, the Netherlands

## Abstract

Emerging viral infections can be identified by using a viral metagenomics approach for clinical human material. Diarrhea samples of patients with unexplained gastroenteritis from the Netherlands were analyzed by using viral metagenomics. Novel circular DNA viruses, bufaviruses, and genogroup III picobirnaviruses were identified. These data expand our knowledge of the human virome.

The list of emerging viral pathogens is ever-changing. The recognition that an increasing number of diseases that were once unexplained are caused by infectious agents has increased substantially in recent years because of breakthroughs in the metagenomics field (*1*). The human gut is a reservoir of a wide variety of microorganisms. In industrialized countries, diarrheal diseases are a major cause of illness among persons of all age groups, and most gastroenteritis cases are caused by viruses (*2*). However, despite extensive diagnostic analysis, the cause of many diarrhea cases remains unresolved.

We analyzed stool samples from 27 patients in the Netherlands who had acute gastroenteritis of unknown etiology for (un)known viruses by using a metagenomics approach. Samples were obtained from patients with sporadic cases and from patients involved in outbreaks of diarrhea and vomiting, for which most common causes of gastroenteritis had been ruled out.

## The Study

Thirteen diarrhea stool samples were obtained from patients with gastroenteritis during 2005–2009 whose infection was not identified despite extensive testing at the reference laboratory for viral gastroenteritis at the National Institute for Public Health and the Environment, Bilthoven, the Netherlands (*3*). In addition, we obtained 14 stool samples from patients hospitalized during 7 gastroenteritis outbreaks in 2008 and 2009 (Table) (*4*). All procedures were performed in compliance with relevant laws (Medical Ethical Committee, University Medical Center Utrecht approval no. 07–310). Samples were analyzed by using a viral metagenomics approach and 169,305 trimmed reads were characterized according to BLAST searches as described (*5*).

**Table:** Detection of mammalian viral sequences in 27 patients with diarrhea by using viral metagenomics, the Netherlands, 2005–2009*

Patient no.	Age, y	Diarrhea type or source	Year of sampling	No. trimmed reads	No. trimmed viral reads	Virus species (no. reads; % nucleotide identity)
VS6600008	7	Sporadic	2008	7,851	271	Human picobirnavirus (221; NA)
VS6600009	25	Sporadic	2008	8,079	10	Bufavirus 1 (7; 67–94)
VS6600010	87	Sporadic	2008	3,237	1	NA
VS6600011	66	Sporadic	2008	3,866	6	NA
VS6600013	48	Sporadic	2008	2,849	19	NA
VS6600014	40	Sporadic	2008	8,079	143	Aichivirus (139; 98)
						Human herpesvirus 4 (1; 91)
						Anellovirus (3; 61–85)
VS6600015	84	Sporadic	2008	11,197	9	Anellovirus (6; 81–97)
VS6600016	37	Sporadic	2008	7,333	1	NA
VS6600017	62	Sporadic	2008	546	0	NA
VS6600018	<1	Sporadic	2008	3,936	4	NA
VS6600019	30	Sporadic	2008	9,590	18	NA
VS6600020	57	Sporadic	2009	10,389	37	Porcine picobirnavirus (6; 57–80)
VS6600021	52	Sporadic	2009	4,587	0	NA
VS6600022	27	OB2005111	2005	4,877	113	Fur seal–associated circular DNA virus (98; NA);
VS6600023	47	OB2005111	2005	7,423	117	Human picobirnavirus (91; 82–87)
VS6600024	47	OB2005115	2005	6,852	338	NA
VS6600025	6	OB2005115	2005	8,949	34	Human picobirnavirus (23; 75–84)
VS6600026	12	OB2006097	2006	6,879	52	Otarine picobirnavirus (42; 70–88)
VS6600027	13	OB2006097	2006	9,481	32	NA
VS6600028	52	OB2008169	2008	5,222	74	NA
VS6600029	32	OB2008169	2008	1,568	4	Human picobirnavirus (3; 86)
VS6600030	26	OB2008190	2008	7,377	57	NA
VS6600031	10	OB2008190	2008	4,541	14	NA
VS6600032	90	OB2008217	2008	4,185	13	Fur seal–associated circular DNA virus (4; NA)
VS6600033	97	OB2008217	2008	3,299	11	NA
VS6600034	89	OB2009024	2009	14,797	168	Human picobirnavirus (115;68%–77%)
VS6600035	91	OB2009024	2009	2,256	8	Human picobirnavirus (3; 70%)

*Classification of sequences was based on taxonomic origin of the best-hit sequence and was performed by using MEGAN version 4.40.4 (http://ab.inf.uni-tuebingen.de/software/megan/) with E cutoff values of 0.001 and 10^–10^ for BLASTn and BLASTx searches (http://blast.ncbi.nlm.nih.gov/Blast.cgi), respectively (*5**,**6*). NA, not applicable; OB, outbreak

Mammalian viral sequences were detected in stool samples from 13 of 27 patients (Table). Anelloviruses that displayed ≈60%–91% nt identities with known anelloviruses were obtained from patients VS6600014 and VS6600015. Because anelloviruses are endemic worldwide, present in many different tissues, and were found in ≈0.05% of the total number of reads, we did not consider it likely that they played a causative role in the gastroenteritis of the patients. Patient VS6600014 was infected with human herpesvirus 4 and an aichivirus; the aichivirus is associated with diarrhea (*6*) and constituted ≈1.7% of the total number of reads. A partial viral protein 2 nucleotide sequence (336 bp covered by 7 reads; KJ206565) of a bufavirus was detected in patient VS6600009. This sequence, which aligned with corresponding sequences of a recently described bufavirus in children with diarrhea in Burkina Faso (*7*), was phylogenetically analyzed and showed 67%–73% nt identity (Technical Appendix Figure 1). Attempts to obtain more sequences from this virus were unsuccessful, and results for real-time PCRs specific for a nonstructural protein 1 gene remained negative, probably because of low virus titers in the sample. New picobirnaviruses and circular DNA viruses were identified and further characterized.

**Figure 1:**
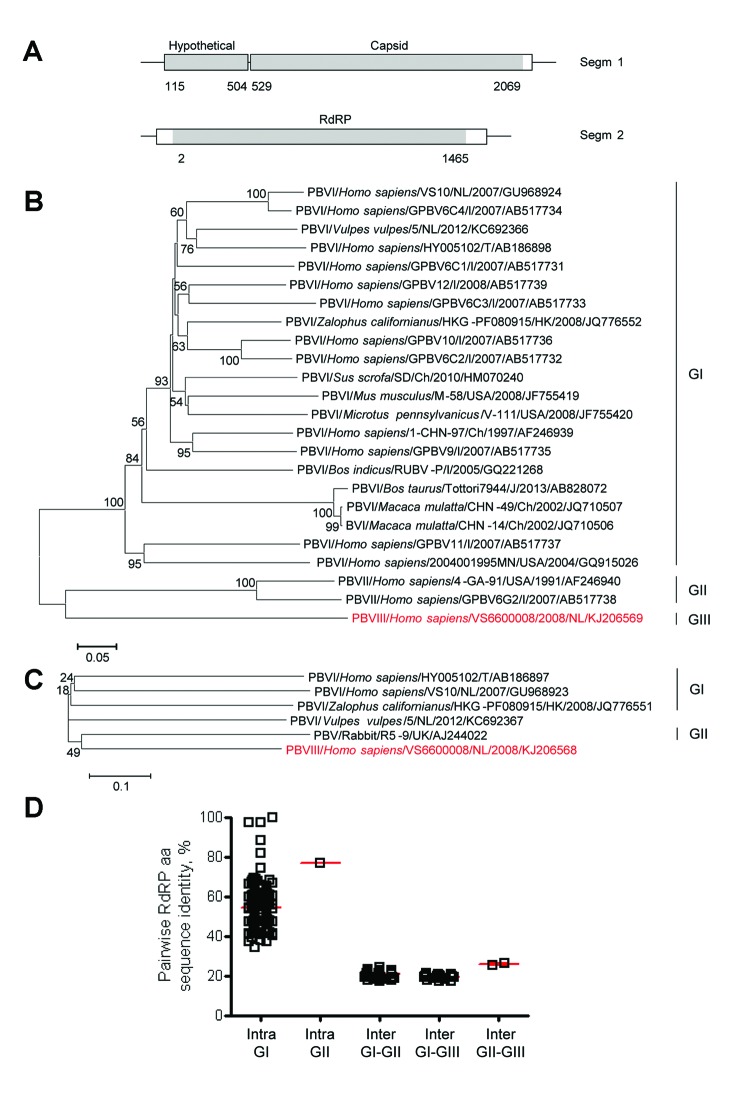
Genome organization and phylogenetic analysis of human picobirnavirus (PBV) VS6600008 isolated in the Netherlands, 2005–2009. A) Putative schematic genome organization of human PBV VS6600008. Locations of major open reading frames are indicated in white and sequences obtained by next-generation sequencing are indicated in gray. Segm, segment; RdRP, RNA-dependent RNA polymerase. B) Phylogenetic neighbor-joining tree with p-distances and 1,000 bootstrap replicates of amino acid sequences of partial RdRP genes corresponding to aa 80–377 of reference PBV strain HY005102; AB186898, PBV VS6600008, and representative PBVs. Alignments were created by using ClustalX 2.0 (http://www.clustal.org/). Viruses are shown as virus/host species/strain/country/year/GenBank accession no. (if available). Virus isolated in this study is indicated in red. Genogroups are indicated on the right. Scale bar indicates amino acid substitutions per site. NL, the Netherlands; I, India; T, Thailand; Ch, China; USA, United States; UK, United Kingdom; J, Japan; HK, Hong Kong. C) Phylogenetic neighbor-joining tree with p-distances and 1,000 bootstrap replicates of the amino acid sequences of the partial capsid genes corresponding to aa 1–220 of reference PBV strain HY005102; AB186897, PBV VS6600008, and representative PBVs. Alignments were created by using ClustalX 2.0. Virus isolated in this study is indicated in red. Genogroups are indicated on the right. Scale bar indicates amino acid substitutions per site. D) Pairwise intragenogroup (Intra) and intergenogroup (Inter) amino acid sequence identities determined by using Bioedit 7.0.9.0 (http://www.mbio.ncsu.edu/bioedit/bioedit.html) between the partial RdRP sequences (corresponding to amino acids 80–377 of reference PBV strain HY005102; AB186898). Each square represents pairwise RdRP amino acid sequence identity between viruses in panel B. Red bars indicate mean and SEM.

Picobirnaviruses are highly variable, double-stranded RNA viruses with a bisegmented genome. Segment 1 (2.2–2.7 kb) encodes the capsid (Cap) protein and potential hypothetical protein(s), and segment 2 (1.2–1.9 kb) encodes the RNA-dependent RNA polymerase (RdRP). On the basis of sequence diversity in RdRP, picobirnaviruses are classified into 2 genogroups (*8*). They have been detected in humans and a wide range of animals (*8*) and might be opportunistic enteric pathogens (*8*,*9*). Stool samples from 7 patients had virus sequences with relatively high homology with known group I picobirnaviruses (Table; Technical Appendix Figure 2). A near-complete highly divergent picobirnavirus genome was obtained by 454-sequencing (Roche, Basel, Switzerland) of samples from patient VS6600008 (GenBank accession nos. KJ206568 and KJ206569). The genome organization is highly similar to that of picobirnaviruses (Figure 1, panel A).

**Figure 2:**
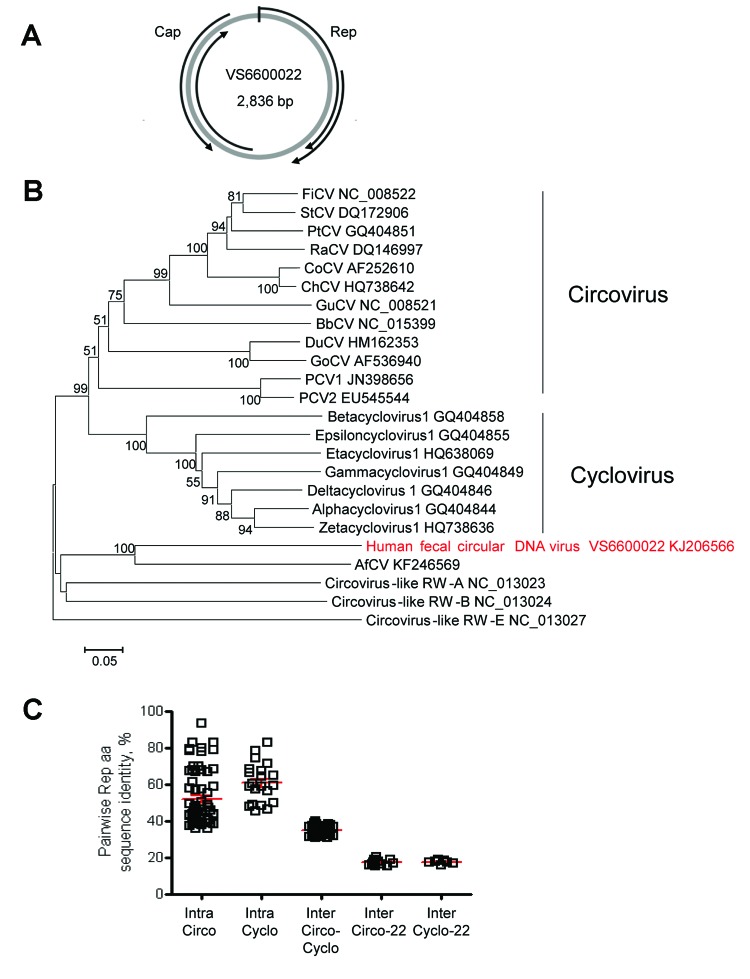
Genome organization and phylogenetic analysis of human fecal circular DNA virus VS6600022, the Netherlands, 2005–2009. A) Putative schematic genome organization. Arrows indicate major open reading frames. Cap, capsid; Rep, rolling circle replication initiator protein. B) Phylogenetic neighbor-joining tree with p-distances and 1,000 bootstrap replicates created with MEGA5 of amino acid sequences of the Rep genes of human fecal circular DNA virus VS6600022 and representative circoviruses that were aligned by using ClustalX2.0 (http://www.clustal.org/). Virus isolated in this study is indicated in red. Scale bar indicates amino acid substitutions per site. FiCV, finch circovirus; StCV, starling circovirus; PtCV, *Pan troglodytes* circovirus; RaCV, raven circovirus; CoCV, columbid circovirus; ChCV, chicken circovirus; GuCV, gull circovirus; PCV, porcine circovirus; BbCV; *Barbus barbus* circovirus; DuCV, duck circovirus; GoCV, goose circovirus; AfCV*, Arctocephalus forsteri* circovirus. C) Pairwise intraspecies (Intra) and interspecies (Inter) amino acid sequence identities determined by using Bioedit 7.0.9.0 (http://www.mbio.ncsu.edu/bioedit/bioedit.html) between the Rep protein sequences of VS6600022 and representative species in the genera *Circovirus* (Circo) and *Cyclovirus* (Cyclo). Each square represents the pairwise RdRP amino acid sequence identity between the viruses in panel B. Red bars indicate mean and SEM.

The pairwise amino acid identity of the partial RdRP of the human picobirnavirus PBVIII/*Homo sapiens*/VS6600008/2008/NL/KJ206569 and that of other representative picobirnaviruses was determined (Figure 1, panels B–D). The intragenogroup amino acid identity between picobirnavirus species ranged from 34.5% to 99.7% in RdRP (Figure 1, panel D). The intergenogroup amino acid identity between genogroup I and II picobirnaviruses ranged from 17.5% to 24.1% (Figure 1, panel D). PBVIII/*Homo sapiens*/VS6600008/2008/NL/KJ206569 showed low amino acid identity (19.4%–26.1%) in the intergenogroup range with genogroup I and II picobirnaviruses (Figure 1, panels B–D), which justifies the placement of this virus in a new genogroup III. Only a few Cap sequences of picobirnaviruses are available; these sequences show <25% amino acid identity to each other, and a clear genogroup division cannot be distinguished (Figure 1, panel C). A picobirnavirus VS6600008-specific real time PCR was performed on the total sample set with primers VS791 (5′-CGATGGATCTTTATGTTCCCG-3′), VS792 (5′-GTAGTTGAAATGTTGATCCATTT-3′), and VS793 (5′-CAAACTTTCCAGCAACCGCTT-3′) labeled with 6-carboxy-fluorescein and 6-carboxy-tetramethyl-rhodamine as described (*10*). Only the sample from patient VS6600008 had a positive result (cycle threshold 25.1).

Novel circular small DNA viruses containing a rolling circle replication initiator protein gene (Rep) have been discovered at increasing rates from animals and humans (*11*). These viruses are extremely diverse and encode at least Cap protein and Rep protein located in opposite genomic orientations and separated by 2 intergenic regions. On the basis of genome organization and amino acid sequence identity of Rep proteins, novel circular DNA viruses seem most closely related to others viruses of the family *Circoviridae* (*11*). A complete circular virus genome (2,836 nt) was obtained from patient VS6600022 by rolling circle amplification and 454-sequencing (KJ206566) (Table). The genome showed an ambisense organization and 2 major inversely arranged open reading frames encoding the Rep and Cap proteins (Figure 2, panel A). A stem-loop structure with the conserved circovirus nonanucleotide motif (5′-TAGTATTAC-3′) was found in the 5′-intergenic region. However, genome size, presence of 2 putative other open reading frames with no sequence homology to any sequence in GenBank, and deviations in WWDGY, DDFYGW, DRYP, FTLNN, TPHLQG, and CSK motifs in the Rep protein, which are ordinarily conserved, indicate that this virus is different from characteristic circoviruses.

Pairwise amino acid identity between the Rep protein of virus VS6600022 and other representative circovirus-like viruses was determined, and a phylogenetic tree was generated (Figure 2, panel B and C). The Rep protein of VS6600022 showed <20% aa identity with all circoviruses and was most closely related to a circular DNA virus from feces of a New Zealand fur seal (33% identity) (*12*). A similar phylogenetic relationship was observed in the Cap protein.

A partial viral genome was identified in patient VS6600032 (KJ206567), and the partial Rep protein of this virus was most closely related to that of VS6600022 (45% aa identity). The cellular host for the novel circular DNA viruses from patients with diarrhea cannot be deduced, and although replication in human cells is conceivable, these viruses might also originate from the diet of the patient. A VS6600022-specific real-time PCR was performed on the total sample set with primers VS794 (5′-ATCGAAGRWCAYCCTGGAAC-3′), VS795 (5′-TKRCACAGGGTACTTGTATC-3′), and VS796 (5′-ACTGTCCTCGTGTACATTGGCAA-3′) labeled with 6-carboxy-fluorescein and 6-carboxy-tetramethyl-rhodamine as described (*13*). Only the sample from patient VS6600022 had a positive result (cycle threshold 34.8).

## Conclusions

Viral metagenomics of patients samples from unexplained diarrhea cases in Netherlands identified viruses of the families *Anelloviridae*, *Picobirnaviridae*, *Herpesviridae*, and *Picornaviridae*, some of which might be associated with development of gastroenteritis (*6*–*8*,*14*,*15*). The discoveries of a new genogroup III picobirnavirus and circular DNA virus from human diarrhea samples expands our knowledge of virus diversity in the human gut. We also showed that recently identified bufaviruses are present beyond the boundaries of Africa (*7*). Mammalian viral sequences were detected in patients with sporadic gastroenteritis and in persons during outbreaks in relatively equal proportions. In addition, specific viral infections were not identified in samples from the same gastroenteritis outbreaks. On the basis of these findings, we cannot conclude or rule out that these viruses cause disease. Further studies are needed to clarify the epidemiology and possible pathogenicity of these viruses in humans.

Technical Appendix Phylogenetic analysis of human bufavirus VS6600009 and a subset of human picobirnaviruses with relatively high homology with known picobirnaviruses, the Netherlands.
