# Supplementary material for: New Viruses in Idiopathic Human Diarrhea Cases, the Netherlands
Source: Emerg Infect Dis. 2014 Jul;20(7):1218–22. doi: 10.3201/eid2007.140190 (PMC4073879; doi:10.3201/eid2007.140190)
Supplement: Technical Appendix — Phylogenetic analysis of human bufavirus VS6600009 and a subset of human picobirnaviruses with relatively high homology with known picobirnaviruses, the Netherlands. [file 14-0190-Techapp-s1.pdf]

Article DOI: http://dx.doi.org/10.3201/eid2007.140190

New Viruses in Idiopathic Human Diarrhea Cases, the Netherlands

Technical Appendix

Phylogenetic analysis of human bufavirus VS6600009 and a subset of human picobirnaviruses with relatively high homology with known picobirnaviruses, the Netherlands.

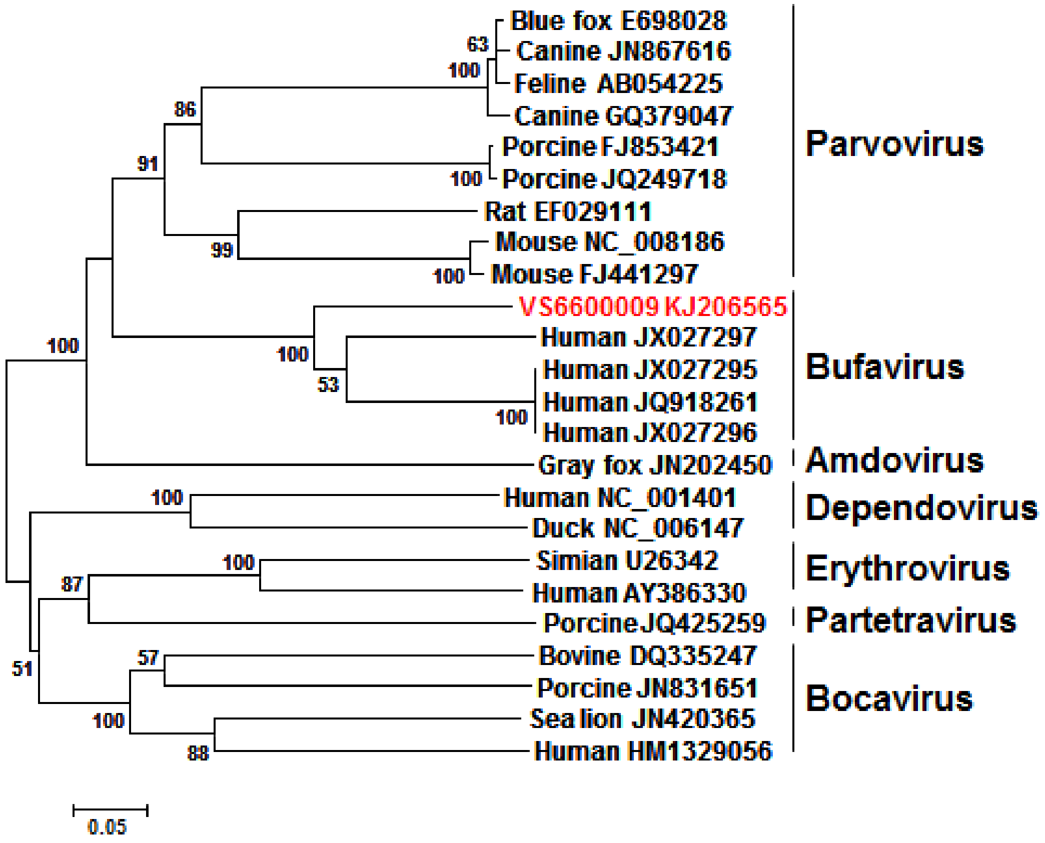

Technical Appendix Figure 1. Phylogenetic analysis of human bufavirus VS6600009 isolated in the Netherlands, 2005–2009. Nucleotide sequences of partial viral protein 2 genes corresponding to nucleotides 3980–4333 of reference human parvovirus B19 (AY386330), bufavirus VS6600009, and representative parvoviruses were aligned by using ClustalX 2.0 (www.clustal.org/). Phylogenetic neighbor-joining tree with p-distances and 1,000 bootstrap replicates was created by using MEGA5 (www.megasoftware.net/). Virus genera, host species, and GenBank accession numbers are indicated. Sequence identities were determined by using Bioedit 7.0.9.0 (www.mbio.ncsu.edu/bioedit/bioedit.html). Virus isolated in this study is indicated in red. Scale bar indicates nucleotide substitutions per site.

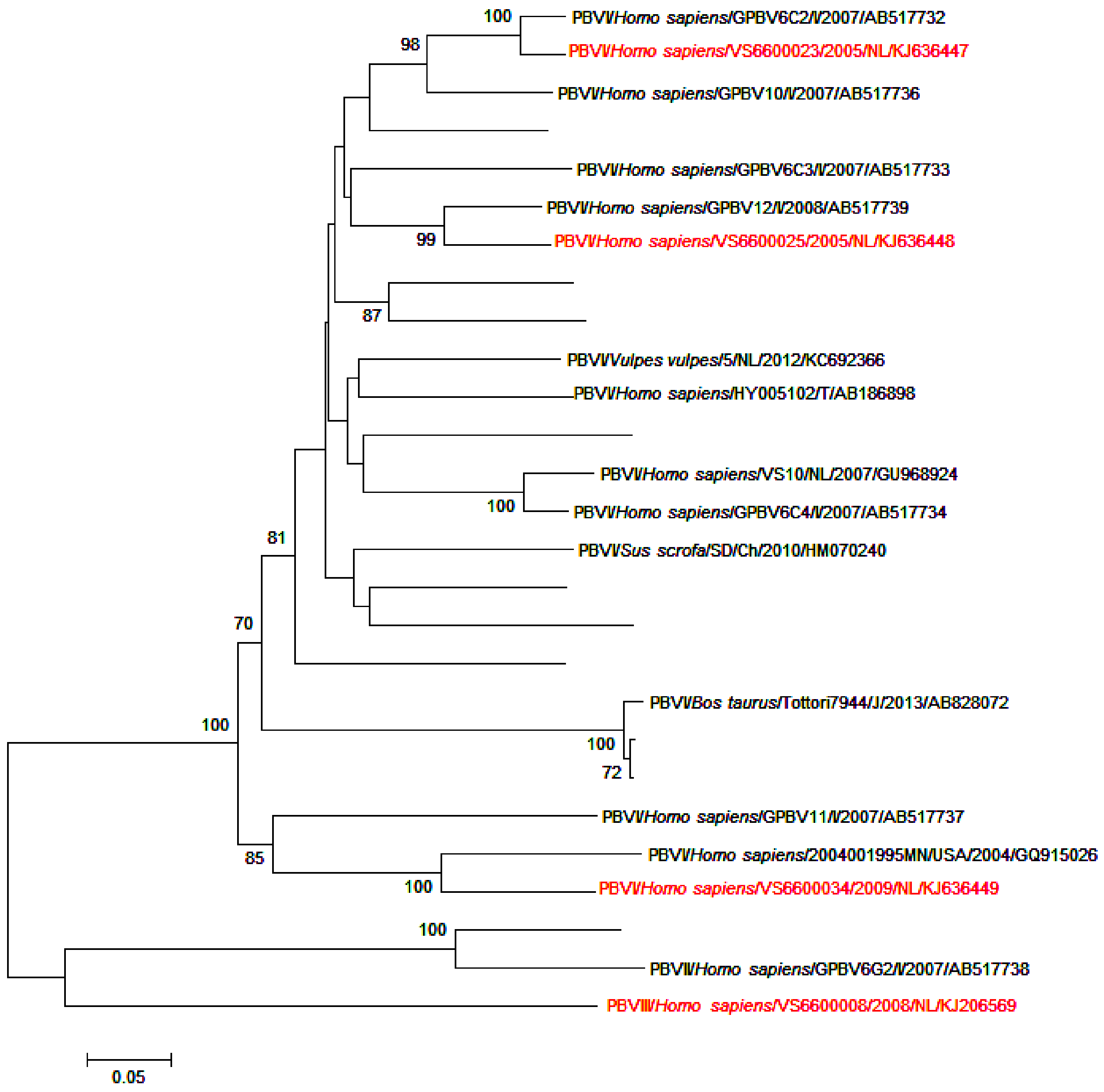

Technical Appendix Figure 2. Phylogenetic analysis of a subset of human picobirnaviruses (PBVs) with relatively high homology with known PBVs, the Netherlands, 2005–2009. Phylogenetic neighbor-joining tree with p-distances and 1,000 bootstrap replicates of amino acid sequences of partial RNA-dependent RNA polymerase genes corresponding to amino acids 200–430 of reference PBV strain HY005102 (AB186898); PBVs VS6600023, VS6600025, and VS6600034; and representative PBVs. Viruses are shown as virus/host species/strain/country/year/GenBank accession number (if available). Viruses isolated in this study are indicated in red. Scale bar indicates nucleotide substitutions per site. NL, Netherlands; I, India; T, Thailand; Ch, China; USA, United States; UK, United Kingdom; J, Japan; HK, Hong Kong.
